# Intervention fidelity and its determinants of focused antenatal care package implementation, in south Wollo zone, Northeast Ethiopia

**DOI:** 10.1186/s12884-021-03637-4

**Published:** 2021-02-19

**Authors:** Asressie Molla Tessema, Abebaw Gebeyehu, Solomon Mekonnen, Kassahun Alemu, Zemene Tigabu

**Affiliations:** 1 Institute of Public Health, University of Gondar, Gondar, Ethiopia; 2 Department of Human Nutrition, Institute of Public Health, University of Gondar, Gondar, Ethiopia; 3 Department of Epidemiology and Biostatistics, Institute of Public Health, University of Gondar, Gondar, Ethiopia; 4 Department of Pediatrics and Child Health, University of Gondar, Gondar, Ethiopia

**Keywords:** Focused antenatal care packages, Intervention fidelity, Neonatal mortality, Implementation of facilitation strategies

## Abstract

**Background:**

Focused antenatal care is directed at sustaining maternal health and improving fetal wellbeing to ensure birth of a healthy neonate. Failure to implement focused antenatal care can result in inability to reduce maternal and perinatal morbidity and mortality in low income countries. Due to evidence-practice gaps, however, thousands of maternal, fetal and neonatal lives are still lost every day, mostly from preventable causes. This study aimed to assess focused antenatal care package’s intervention fidelity and its determinant factors in South Wollo Zone, Northeast Ethiopia.

**Methods:**

A cross-sectional study design was employed and a total of 898 women who gave birth in the last 6 months prior to data collection were included. Also 16 health extension workers, working in ten selected health posts, were included. Interviews and self-administered questionnaires were used to collect data from mothers and health extension workers. Ten [10] health posts were audited to assess availability and functionality of drugs and supplies to provide focused antenatal care. Mothers were asked whether or not the required level of care was provided. Health extension workers were provided with self-administered questionnaires to assess socio-demographic characteristics, reception of training, facilitation strategies for the implementation of focused antenatal care and ability to classify danger signs. Multilevel linear regression analysis was performed to identify individual and organizational level’s factors influencing focused antenatal care package intervention fidelity.

**Results:**

Overall weighted average focused antenatal care package intervention fidelity (implemented as intended/planned) was 49.8% (95% CI: 47.7–51.8), which means the average number of focused antenatal care package interventions women received is 49.8%. Health extension workers implemented 55.1% and skilled providers (nurses, midwives, health officers or medical doctors) 44.9% of focused antenatal care package interventions. Overall antenatal care coverage, irrespective of frequency (at least one visit), was 752/898 women (83.7%; 95% CI: 81.3–86.1); 263/752 women (35.0%; 95% CI: 31.6–38.4) received at least four antenatal visits and only 46/752 women (6.1%; 95% CI: 4.4–7.8) received all recommended components of focused antenatal care. Previous pregnancy-related problems, paternal education and implementation of facilitation strategies were found to be significant factors enhancing focused antenatal care package intervention fidelity.

**Conclusion:**

Focused antenatal care package intervention fidelity in the study area was low; this may imply that the current level of maternal, perinatal and neonatal mortality might be partly due to the low level of focused antenatal care intervention fidelity. Improving implementation of facilitation strategies is highly required to contribute to the reduction of those mortalities.

## Background

Focused antenatal care (FANC) is evidence based, women oriented, goal directed and individualized care for pregnant women to improve maternal, perinatal and neonatal outcomes. FANC includes clinical assessment of pregnant women and their fetus during pregnancy in order to achieve favorable outcomes for both women and fetus. FANC’s interventions include identification and management of obstetric complications and infections, promoting using skilled attendants and healthy behavior [1]. FANC activities are directed at sustaining maternal health and improving fetal wellbeing to ensure birth of healthy neonates. Failure to implement FANC can result in inability to reduce maternal, perinatal and neonatal morbidity and mortality in low income countries [2, 3].

Evidence shows that public health interventions during the antenatal period are effective to reduce maternal, perinatal and neonatal mortality [4]. In all studies reviewed here, interventions during pregnancy significantly reduced neonatal mortality in addition to improving fetal and maternal health. Studies conducted in Indonesia, Bangladesh, sub-Saharan Africa and India indicated that increasing the number of antenatal visits has shown to decrease maternal, perinatal and neonatal mortality [5–8]. Several studies demonstrated that prenatal iron and folic acid supplementation [9–15], tetanus toxoid vaccination [9, 16–19], use of insecticide-impregnated bed nets during pregnancy [4] and syphilis screening and treatment [15] have shown to reduce maternal, perinatal and neonatal mortality. Importantly, randomized trials and large observational studies showed significant reductions in neonatal mortality and improvement of maternal and childcare uptake after implementing these interventions as a package in community settings [4, 20–27]. The bare number of antenatal visits does not have a significant reducing effect on those mortalities [22].

Ethiopia is implementing FANC package in community settings since 2013 to achieve a reduction in maternal, perinatal and neonatal mortality [28]. One-to-five networks are a household-based government strategy, consisting of one leader with five member households for reaching women and their children. Five to six of such networks can make one-to-thirty health development army (HDA) teams. HDA is an innovative, inclusive and collaborative strategy of Ethiopian government composed of 25–30 unpaid women volunteers in neighboring households [29–32]. It aims at early identification of pregnant women and provision of FANC by linking community levels of care with health extension workers (HEWs) in community health posts to primary health care units. Even after the introduction of FANC, however, maternal and child health indicators in Ethiopia are still among the highest in the world. The main question here is, why those maternal, neonatal and child health indicators remained high while these effective intervention packages are implemented? We hypothesize that these interventions may not be properly implemented as per standard, commonly known as evidence-practice gaps. To our knowledge, no study thus far assessed whether FANC is implemented with fidelity or not, and facilitators enhancing and barriers inhibiting FANC intervention fidelity influenced its implementation. Intervention fidelity refers to the degree to which interventions are implemented as planned in the original implementation document [33]. Therefore, FANC package intervention fidelity is defined as the degree to which the FANC package is implemented as described by community-based newborn care (CBNC) plan, which was developed by the Ministry of Health of Ethiopia. This study aimed to assess FANC package intervention fidelity and its determinants in South Wollo Zone, Northeast Ethiopia.

## Methods

### Design

Cross-sectional study design was used for evaluating intervention fidelity of FANC package in south Wollo Zone, North east Ethiopia.

### Context

FANC package is a combined effective and efficient public health intervention provided at household, health post, health center and hospital levels. Main implementers are HDAs, HEWs, and skilled health providers in health centers. HEWs, who are young females with 10th grade education completed, have been trained and certified to provide family health care at community level, including FANC, diseases prevention and control, hygiene and environmental sanitation, health education and communication [28, 34, 35]. HEWs work in health posts under supervision and support of health centers.

### Targeted sites and populations

The study was conducted in South Wollo Zone of the Amhara region, which is 400 kms north of Addis Ababa, capital of Ethiopia. There were 900 rural and 150 urban HEWs, 499 health posts, 126 health centers and 9 hospitals (one zonal) in the Zone. All mothers who gave birth in the last 6 months of data collection, HEWs and health posts in the selected kebeles’ were included in the study. In Ethiopia, Kebele is the smallest administrative unit.

### Intervention description

FANC requires a continuum of care provided at household, health post, health center and hospital levels. Main goal of the intervention package is to transform evidence into action for reducing maternal, perinatal and neonatal morbidity and mortality by increasing the reach to all pregnant women and newborns in the community. FANC includes provision of four antenatal visits, counseling on nutrition, impregnated bed net use, danger signs and mother to child HIV-transmission. It also includes birth preparedness and complication readiness planning, treatment of diagnosed sexually transmitted infections (STI), blood pressure, height and weight measurement in addition to identification of maternal danger signs and referral if necessary, provision of two doses of tetanus toxoid vaccination, promotion of facility birth, iron and folate supplementation and detection and management of complications. Facilitation strategies include weekly supervision and support of HEWs by health center staff, monthly supervision by Woreda health office, community and HDA support [28].

### Subgroup (sampling)

Kebeles from South Wollo Zone were selected randomly using computer-generated random numbers. All mothers who gave birth in the last 6 months (for individual-level variables and fidelity assessment), all HEWs and all health posts in the selected kebeles (for cluster-level variables) were included. Mothers were interviewed in their homes and HEWs completed the questionnaires by themselves while their facility was audited. Facility audit is a review of a facility’s assets, important for provision of FANC.

### Outcomes

Primary outcome of this study, FANC package intervention fidelity was computed by the weighted average of program reach (contact coverage), adherence to FANC contents and frequency. Program reach was measured by the proportion of mothers who visited any health facility at least once and provided by any health care provider during recent pregnancy. The number of antenatal visits and components provided for the mothers were considered as frequency and content.

### Sample size determination

Considering 52% of pregnant mothers who received 4+ antenatal care visits and all contents of antenatal care, 95% confidence level, 5% margin of error with 10% non-response rate, 422 participants were required [36]. However, due to cluster sampling of kebeles, we collected data from 898 mothers. In addition, sixteen HEWs were included, and ten health posts where those 16 HEWs worked, were audited.

### Statistical analysis

Antenatal care coverage, frequency and content were computed by considering the recommended amount of FANC as a reference. Antenatal care coverage was determined as the proportion of women who have been contacted at least once by health care providers during pregnancy. Since the recommended number of FANC visits was at least four, getting antenatal care frequency less than four was weighed (as $\frac{1}{4},\frac{2}{4},\frac{3}{4},\frac{>4}{4}$) by considering > four as one (reference). For antenatal care contents, taking the total 17 antenatal care issues as maximum (Table 1), mothers who received less than the recommended contents during pregnancy were weighted accordingly (as $\frac{1}{17},\frac{2}{17},\frac{3}{17},\frac{4}{17},\dots \frac{17}{17}$). As there was no previous study that assessed intervention fidelity, equal weights were given for coverage, components and frequency to compute fidelity [37, 38]. FANC package intervention fidelity was calculated by taking the mean of the weighted product of antenatal care coverage, frequency, and contents. Health posts were audited for the presence and functionality of supplies and equipment necessary for FANC. Multilevel statistical model was considered because mothers were nested from the health post and the sampling method was cluster sampling (by kebeles). Before jumping to multilevel model, intra-cluster correlation coefficients (ICC) were computed and > 5% was used as a cutoff point. Exploratory data analysis was performed using SPSS version 20 and statistical modeling was conducted by R statistical software. Both Akaike’s Information Criteria (AIC) and Bayesian Information Criteria (BIC) were used for checking model fitness. This study used standards for reporting implementation research guidelines (StaRI) [33].

**Table 1:** Components of focused antenatal care package provided by HEWs and skilled providers’ qualification in South Wollo Zone, Ethiopia

Contents		Number of mothers (%)	By HEWs (%)	By skilled provider (%)
*Weight measured*	Yes	629 (83.6)	296 (47.1)	333 (52.9)
	No	123 (16.4)	101 (82.1)	22 (17.9)
*Height measured*	Yes	488 (64.9)	252 (51.6)	236 (48.4)
	No	264 (35.1)	145 (54.9)	119 (45.1)
*Blood pressure measured*	Yes	558 (74.2)	305 (54.7)	253 (45.3)
	No	194 (25.8)	92 (47.4)	102 (52.6)
*Advised for institutional birth*	Yes	686 (91.2)	372 (54.2)	314 (45.8)
	No	66 (8.8)	25 (37.9)	41 (62.1)
*Advised for BPCR*^a^	Yes	645 (85.8)	350 (54.3)	295 (45.7)
	No	107 (14.2)	47 (43.9)	60 (56.1)
*Advised on danger signs during pregnancy and birth*	Yes	606 (80.6)	327 (54.0)	279 (46.0)
	No	146 (19.4)	70 (47.9)	76 (52.1)
*Advised on personal hygiene*	Yes	695 (92.4)	372 (53.5)	323 (46.5)
	No	57 (7.6)	25 (43.9)	32 (56.1)
*Advised for PMTCT*^a^	Yes	633 (84.2)	320 (50.6)	313 (49.4)
	No	119 (15.8)	77 (64.7)	42 (35.3)
*Advised and screened for STI*^a^	Yes	622 (82.7)	308 (49.5)	314 (50.5)
	No	130 (17.3)	89 (68.5)	41 (31.5)
*Advised for bed net use*	Yes	527 (70.1)	299 (56.7)	228 (43.3)
	No	225 (29.9)	98 (43.6)	127 (56.4)
*Mothers tested for HIV*	Yes	678 (90.2)	345 (509)	333 (49.1)
	No	74 (9.8)	52 (70.3)	22 (29.7)
*Advised for nutrition during pregnancy*	Yes	634 (84.3)	350 (55.2)	284 (44.8)
	No	118 (15.7)	47 (39.8)	71 (60.2)
*Told to seek care for pregnancy danger signs*	Yes	669 (89.1)	363 (54.3)	306 (45.7)
	No	82 (10.9)	34 (41.5)	48 (58.5)
*Number of TT*^a^ *vaccine received*	No	58 (7.7)	23 (39.7)	35 (60.3)
	TT_1_	287 (38.2)	185 (64.5)	102 (35.5)
	TT_2_+	407 (54.1)	189 (46.4)	218 (53.6)
*Iron folic acid received*	Yes	425 (56.5)	203 (47.8)	222 (52.2)
	No	327 (43.5)	194 (59.3)	133 (40.7)
*Referred for institutional birth*	Yes	475 (63.2)	284 (59.8)	191 (40.2)
	No	277 (36.8)	113 (40.8)	164 (59.2)
*Expected date of birth told*	Yes	462 (61.4)	228 (49.4)	234 (50.6)
	No	290 (38.6)	169 (58.3)	121 (41.7)

^a^*BPCR* Birth preparedness & complication readiness, *PMTCT* Prevention of mother to child transmission of HIV, *STI* Sexually transmitted infection, *TT* Tetanus toxoid

## Results

### Socio-demographic characteristics

Mean age of women at the time of interview was 30.96 + 7.22 years. Of 898 women, 449 (50%) were between 25 and 36 years of age. Six hundred thirty eight (71.4%) did not attend any formal education, 768 (85.5%) were married and 662 (74%) of them were housewives.

HEWs’ mean age was 26 + 3.67 years and 13 (81.3%) of them were married and they walked an average of around 3 h (95% CI: 2:05–3:05) to reach to the most far away mother’s home.

### Coverage of FANC

Seven hundred fifty two of 898 women were contacted by health care providers at least once during their recent pregnancy, making an overall antenatal care coverage of 83.7% (95% CI: 81.28–86.12). Out of those 752 women, ANC was provided by HEWs for 397 (52.8%; 95% CI: 52.7–52.9) and for 355 (47.2%; 95% CI: 47.1–47.3) by skilled providers. Mean time of first antenatal care visit was 4.14 + 2 months. Interestingly, 344/752 (45.7, 95% CI: 44.1–47.7) attended ANC in the first trimester of gestation (less than 12 weeks).

### Frequency and contents of FANC

**Fig. 1:**
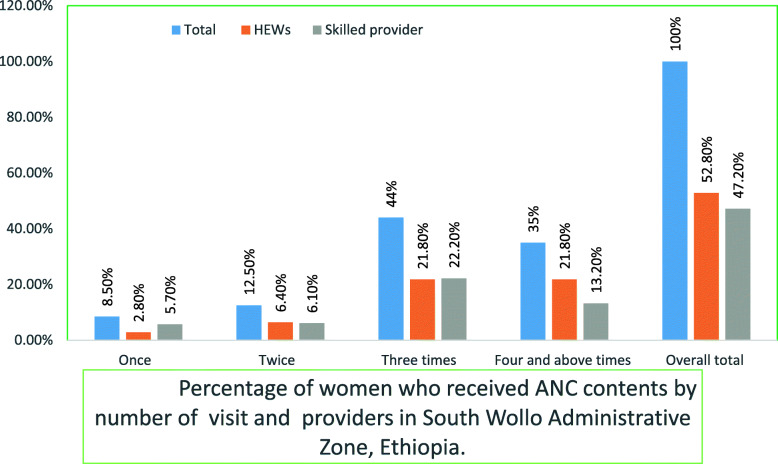
Percentage of women who received ANC contents by number of visit and providers in South Wollo Administrative Zone, Ethiopia

Mean number of antenatal visits were 3 + 1.6, and 263/752 women (35.0%; 95% CI: 31.6–38.4) attended at least four ANC visits. Higher numbers of antenatal visits were related with increased FANC package contents provided to mothers (Fig. 1).

Only 46/752 women (6.1%; 95% CI 4.4–7.8) received all contents of FANC. Thirty three of those (4.3%) received care from HEWs and 13 (1.7%) from skilled providers (Table 1).

Overall weighted average FANC package intervention fidelity (implementation as planned or average number of FANC package interventions women received) was 0.498 (49.8%; 95% CI 47.7–51.8); HEWs provided 0.62 (62.0%; 95% CI: 59.7–64.3) while skilled providers provided 0.566 (56.6%; 95% CI 53.9–59.2). Only 20 women (2.2%) received the whole recommended FANC package with full fidelity.

### Provider-related factors

Twelve (75%) HEWs were ever trained on FANC package while only 2 (12.5%) of them received refreshment training in the last 3 months. Only two health posts were supervised weekly from the catchment health center and 9 (56.3%) HEWs received onsite assistance for difficult cases. Nine of them responded that they were able to provide FANC.

Support strategies set by Ministry of Health were assessed from health center, district health offices, community and development armies’ perspective. Eleven (68.8%) HEWs reported that implementation of support either from the community, health development armies or district health office was lower than the planned standard.

### Women-related factors

Only 180 women (20%) were living within 15-min’ walking distance from the health post, while 333 (37.1%) of them had to travel > 45 min on foot. Of those who received antenatal care, 685 (91.1%) were self-referrals. One hundred eighty seven (20.8%) encountered pregnancy-related problems like bleeding, convulsions or high temperature in their previous pregnancies.

### Organizational-related factors

No health post had all required functional equipment and medical supplies for FANC. Birth preparedness and complication readiness forms, supervision checklists, blood pressure cuffs, pregnant women registration books, stethoscopes and tape measures were the most frequently mentioned unavailable items in health posts.

### Facilitators’ and barriers’ of FANC intervention fidelity

The ICC observed in the model was 17.7%, which indicates that 17.7% of the variation in FANC package intervention fidelity is explained by health post (cluster) level factors. This shows FANC package intervention fidelity varies between health posts and there are health post level factors which affect implementation of the package.

In the first level model, maternal age, distance from the health post, maternal education, pregnancy-related problems in previous pregnancies, partner’s education and total number of abortions were considered. Facilitation strategies, distance from the farthest household and availability of supplies in the health post were considered in the second level model. Finally, pregnancy-related problems in previous pregnancies, partner’s education and facilitation strategies were found to be statistically significant facilitators for FANC package intervention fidelity (Table 2). In this study, women with pregnancy-related problems in previous pregnancies had a 9% increase of FANC package intervention fidelity as compared to those without. Women who had formally educated partners had an 8% increase in the levels of FANC package intervention fidelity in recent pregnancies as compared to their counterparts. An average increased implementation of health post level facilitation strategies resulted in a 4% increase in the level of FANC package intervention fidelity.

**Table 2:** The following table shows the initial (maternal level) and final (combined) variables with the corresponding beta coefficient and confidence intervals of mixed effect model

Variables		Estimate	95% Confidence interval
**Level 1 variables**			
Age of mothers (in years)		0.004	0.0003–0.008
Maternal problems in previous pregnancy	No	1	
	Yes	0.06	0.01–0.11
Total number of abortions		0.01	0.005–0.022
Husband education	No formal education	1	
	Attend formal education (1–8)	0.10	0.05–0.15
**Combined model**			
Pregnancy related-medical problems in previous pregnancy	No	1	
	Yes	0.09	0.02–0.15
Husband education	No formal education	1	
	Attend formal education (1–8)	0.08	0.02–0.13
Implementation of supportive/ facilitation strategy		0.04	0.02–0.05

In the final model, ICC was reduced to 4.7% and both Akaike’s Information Criteria (AIC) and Bayesian Information Criteria (BIC) decreased to 187.3 and 168.6 from the initial model (AIC 334.6 and BIC 349.0).

## Discussion

Antenatal care coverage was 83.7%; 6.1% women received all recommended components and 35% received at least four ANC visits. Moreover, over 90% were self-referrals to antenatal care. The overall weighted average FANC package intervention fidelity was 49.8% and of these, 62.0% were by HEWs and 56.6% by skilled providers. Having pregnancy-related medical problems, formally educated partners and implementation of facilitation strategies were significant facilitators for FANC package intervention fidelity.

In this study, the weighted average FANC package intervention fidelity was too low compared to the standard from the implementation plan. Durlak et.al. suggested that the level of an intervention implementation should be around 60% to produce positive results [39]. This might imply that the FANC package intervention fidelity, according to our findings, is too low to result in an anticipated reduction of maternal, perinatal and neonatal mortality and morbidity. We have also shown that the level of FANC frequency was 35.0% and content of 6.1%. Non-conformity with prescribed frequency (≥4 visits) and recommended components of FANC was extremely evident. In the study, the observed increase in antenatal care visits was accompanied by implementation of more FANC components, which is in line with findings from 41 countries’ demographic and health surveys [40]. Mere increase in the number of ANC visits does not lead to increased provision of expected components and intervention fidelity of the FANC package.

It is indicated in this study that history of pregnancy related problems increases the use of FANC intervention package which is contrary to findings of other studies in Ethiopia that assessed the factors affecting contact coverage [41]. The difference might be due to the difference in outcome of interest as our study is the combination of coverage, frequency and content. This may indicate that mothers’ pregnancy-related risk perceptions play an important role for their adherence to the recommended FANC package interventions.

Partners’ attendance of formal education facilitates FANC package intervention fidelity, contrary to women with the same level of education. Paternal education, even at the lowest level, thus contributes to improved uptake and adherence to the recommended package of care which is in line with another systematic review and primary study [41, 42]. Maternal and child (MCH) health care uptake decision-making may depend on partners, particularly for mothers with low levels of education. Therefore, MCH policy development and implementation needs to involve partners, particularly for mothers with low levels of education in rural areas.

When facilitation strategies, put in place to optimize implementation of FANC package intervention, increased, intervention fidelity of FANC may be optimized to its expected level. When HEWs get support from community, HDA, health center and district health office staff as planned, provision of FANC package intervention will be enhanced. Measuring facilitating effects of supportive strategies is essential for optimizing and harmonizing the FANC package intervention implementation [43]. Weakness in the facilitation strategy could be one of possible reasons for the observed low level of FANC package intervention fidelity, thereby contributing to the high level of maternal, perinatal, neonatal mortality and morbidity in Ethiopia.

Nonetheless, information was collected from the mothers by non-health professional data collectors during the last 6 months that the issue of social desirability bias needs to be considered in interpreting our findings.

## Conclusion

This study showed FANC package intervention fidelity to be low. High levels of maternal, perinatal and neonatal mortality indicators may be partly due to low levels of FANC package intervention fidelity, pointing to implementation problems. Maternal previous pregnancy-related problems, partner’s education and implementation of facilitation strategies were significant facilitators of FANC package intervention fidelity. Paternal education and implementation of facilitation strategies play significant roles for improved FANC package intervention fidelity. Further studies are needed that focus on why facilitation strategies were relatively underused during implementation.

## Data Availability

The dataset used and analyzed for this study is available from the corresponding author. This data can be made available upon reasonable request.

## Abbreviations

sSA: Sub-Saharan Africa
SDG: Sustainable Development Goals
FANC: Focused Antenatal Care
HEWs: Health Extension Workers
CBNC: Community-Based Newborn Care
HDA: Health Development Army
WDA: Women Development Army
KMs: Kilo-Meters
HIV: Human Immunodeficiency Virus
STI: Sexually Transmitted Infection
ICC: Intra-cluster Correlation Coefficient
SPSS: Statistical Package for Social Sciences
AIC: Akaike’s Information Criteria
BIC: Bayesian Information Criteria
StaRI: Standards for reporting implementation research
ANC: Antenatal Care
MCH: Maternal and Child Health
